# Family functioning, trauma exposure and PTSD: A cross sectional study

**DOI:** 10.1016/j.jad.2018.11.056

**Published:** 2019-02-15

**Authors:** Sarah Dorrington, Helena Zavos, Harriet Ball, Peter McGuffin, Athula Sumathipala, Sisira Siribaddana, Fruhling Rijsdijk, Stephani L. Hatch, Matthew Hotopf

**Affiliations:** a Department of Psychological Medicine, Institute of Psychiatry, Psychology and Neuroscience, King's College London, UK; b MRC Social, Genetic and Developmental Psychiatry Centre, Institute of Psychiatry, Psychology and Neuroscience, King's College London, UK; c North Bristol NHS Trust, Bristol, UK; d Department of Primary Care and Health Services, Keele University, Staffordshire, UK; e Faculty of Medicine & Allied Science, Rajarata University of Sri Lanka, Anuradhapura, Sri Lanka; f South London and Maudsley NHS Foundation Trust, UK; g Department of Psychology, Institute of Psychiatry, Psychology and Neuroscience, King’s College London, UK

## Abstract

**Objective:**

Only a minority of trauma-exposed individuals go on to develop post traumatic stress disorder (PTSD). Previous studies in high income countries suggest that maladaptive family functioning adversities (MFFA) in childhood may partially explain individual variation in vulnerability to PTSD following trauma. We test in a lower middle-income setting (Sri Lanka) whether: (1) MFFA is associated with trauma exposure; (2) MFFA moderates the association between exposure to trauma and later (a) PTSD (b) other psychiatric diagnoses; and (3) any association between MFFA and PTSD is explained by experiences of interpersonal violence, cumulative trauma exposure or comorbid psychopathology.

**Methods:**

We conducted a population study of 3995 twins and 2019 singletons residing in Colombo, Sri Lanka. Participants completed the Composite International Diagnostic Interview, including nine traumatic exposures and a questionnaire on MFFA.

**Results:**

23.4% of participants reported exposure to MFFA. We found that (1) MFFA was strongly associated with trauma exposure (2) MFFA moderates the association between trauma exposure and both (a) PTSD and (b) other DSM psychiatric diagnosis. (3) This was not explained by interpersonal violence, cumulative trauma exposure or other psychopathology.

**Conclusions:**

MFFA moderates the association between trauma and PTSD, and the association between trauma and non-PTSD psychopathology.

## Highlights

• We find that people who have more adversity in childhood accumulate more trauma in later life.
• The excess PTSD in people with childhood adversity may partially be explained by greater subsequent trauma exposure, but not by trauma severity or comorbid psychiatric disorder.
• This suggests a specific relationship between childhood adversity and subsequent vulnerability to PTSD.

## 1 Introduction

Cross sectional and cohort studies consistently show that the distribution of post traumatic stress disorder (PTSD) in populations is not solely a function of exposure to trauma (Brewin et al., 2000). Only a minority of individuals exposed to trauma develop PTSD (Creamer et al., 2001, Frissa et al., 2013, Perkonigg et al., 2000) and many contextual variables have been identified which appear to increase risk of PTSD following trauma (Ehlers and Clark, 2000, Iversen et al., 2009). One important risk factor is early adversity, including maladaptive family functioning adversities (MFFA)(Kessler et al., 2010, McLaughlin et al., 2017). These adversities include aspects of parenting, including neglect, antipathy, separation and punishment, and may partially explain individual variation in vulnerability to PTSD (Brewin et al., 2000, Iversen et al., 2007, Kessler et al., 2010, McLaughlin et al., 2017, Ozer et al., 2003).

There are numerous pathways by which an association between MFFA and PTSD vulnerability might operate. Firstly, traumatic events are not randomly distributed within populations, and people with MFFA may experience more, or different, traumatic events than the general population. Stress proliferation theory suggests that MFFA is associated with social deprivation which in turn is associated with greater exposure to traumatic life events (Pearlin, 1989, Pearlin et al., 2005). In addition, MFFA may lead to greater risk-taking in affected individuals, leading again to more traumatic events (Felitti et al., 1998). Secondly, MFFA may act as an effect modifier, by increasing vulnerability to PTSD when traumatic events occur. For example, there is evidence from basic and cognitive neuroscience that exposure to early adversity has far-reaching impacts on brain development and cognitive processes which may reduce resilience in the face of trauma in later life (Bick and Nelson, 2016, Dannlowski et al., 2012, Ferreira et al., 2014, Whittle et al., 2013). Finally, because MFFA are associated with many other psychiatric disorders, and there is a strong relationship between PTSD and other psychiatric disorders, the relationship is confounded by psychiatric comorbidity (Breslau, 2009, Dorrington et al., 2014, Kessler, 1995).

In a previous study we found strong associations between trauma exposure and PTSD, with both the dose and type of trauma, in particular, we found that experience of interpersonal violence was strongly associated with PTSD (Dorrington et al., 2014). However, these associations were not specific to PTSD – the dose and type of trauma were also strongly associated with other psychiatric diagnoses, and collectively these outcomes were considerably more common in the trauma-exposed population than was PTSD.

The vast majority of research on early adversity, trauma and PTSD is based on Western populations (Baxter et al., 2013, Kessler et al., 2010, Norman et al., 2012, Saxena et al., 2006). We here report data based in a low-middle income country, Sri Lanka. Our aims were to explore the impact of MFFA on PTSD. Firstly, we test the hypothesis that MFFA is associated with type and dose of trauma exposure. Secondly, we test whether, given the experience of trauma, individuals with MFFA are more likely to experience (a) PTSD and (b) other psychiatric diagnosis. Lastly, we test whether any such association between MFFA and PTSD in the trauma exposed group can be explained by comorbid psychopathology, cumulative trauma or high-risk trauma (interpersonal violence).

## 2 Method

### 2.1 Study design and participants

The Colombo Twin And Singleton Study (CoTASS) is a population-based twin study with a comparable non-twin sample. Full details of the design and implementation of the study are described elsewhere (Siribaddana et al., 2008). The study took place in the Colombo District of Sri Lanka, an area with a population of 2.2 million which includes the island's capital. The district has a mixture of urban and rural populations with 45% of the population officially designated as living in rural communities (2001).

### 2.2 Inclusion/Exclusion criteria

Individuals were excluded if they failed a mini mental state examination, or where interviews were conducted via a proxy. Twins were excluded if the individuals said they were not twins; one or both of the pair had died or gone abroad; or there were no twins at the given address.

The annual update of the electoral register consists of a household census. We added a question asking whether the householder knew of any twins, and identified 19302 individual twins by this method. Of these, we randomly selected 4387 twins to take part in the present study. Four thousand and twenty-four (91.7%) participated, and interviews were completed for 3995 (including 72 unpaired twins and 5 sets of triplets). Of the 1954 complete pairs, 1420 (72.7%) were same-sex pairs (of which 635 were male–male (44.7%), and 830 were classified as monozygotic (58.5%), and 534 (27.3%) were opposite-sex pairs. In addition, we conducted a parallel study of non-twins, randomly sampled from the same local areas from which twins were recruited. Two thousand three hundred and eleven non-twins were selected and eligible to participate, of whom 2019 (87.4%) consented and were interviewed. The twin and non-twin samples had similar sex profiles, but twins were younger (Siribaddana et al., 2008). We included all consenting individuals aged 15 years or older who spoke sufficient Sinhala to understand the interview. Due to power constraints, analyses based on ethnicity have collapsed participants into Sinhala and non-Sinhala categories.

Interviews took place between 2006 and 2007, when Sri Lanka had been experiencing violent civil war for over 20 years. There had been uprisings and bombing attacks in Colombo, and at times a strong military presence. While many people in Colombo have been indirectly affected by the tsunami of 2004, the district experienced comparatively minor damage and disruption compared to the south of the island.

### 2.3 Data collection

Research workers educated to ‘A’ level standard visited the participants' homes to interview them separately. We used the Composite International Diagnostic Interview (1990)**,** a structured diagnostic interview for use by lay interviewers. We used qualitative techniques to adapt the measures. The measures were sent to a total of 13 bilingual twins (contacted from the registry) and other Sri Lankans fluent in English and Sinhala. Each measure (or in some cases subcomponent of measures) was translated at least twice independently. The translations were then reviewed in group-meetings consisting of seven professionals (6 doctors and one health service researcher, all with a background in mental health). A scholar in Sinhala also checked the translation. The adaptation was not a direct, literal translation, but aimed to find forms of words in Sinhala that best described the concepts of interest and if the questions, when translated, seemed cumbersome, they might be broken down into two component items to improve clarity. The interviews were then trialed by multiple volunteers recruited from field workers and four individuals with no connection to the study, in order to confirm that lay people could understand it.

## 3 Measures

### 3.1 Composite International Diagnostic Interview (CIDI)

The World Health Organisation's CIDI was used to generate lifetime DSM-IV diagnoses of mental disorders (Widiger and Samuel, 2005; World Health Organisation, 1990). The CIDI PTSD module includes a series of questions about lifetime traumas, the DSM Trauma Events Questionnaire. Previous studies have used expanded versions of the Traumatic Events Questionnaire, with up to 36 specified traumatic events (Breslau, 1998, Kessler, 1995, Vries and Olff, 2009). The questionnaires applied in our study, and by Breslau et al. (1991) specify just nine different traumatic events (Table 2) (Breslau et al., 1991, Dorrington et al., 2014).

We used a modified version of the criterion A traumatic events because local experts were concerned about the acceptability of asking about sexual trauma at first contact in a population study. The remaining list of events included nine traumatic, events (physical attack, tortured or terrorised, threatened with weapon or kidnapped, shock of event to someone else, involved in combat, natural disaster, life-threatening accident, witnessed killing or accident, other stressful event). If one or more of these events were endorsed, the rest of the PTSD module was administered. A previous analysis of this population (Dorrington et al., 2014) found three measures of interpersonal violence to have the highest conditional probability of PTSD: ‘tortured or terrorized’, ‘physical attack’ and ‘threatened with a weapon or kidnapped’. These three items are combined in this paper as a measure of high risk trauma exposure ‘interpersonal violence’.

In addition to PTSD, information was collected on affective disorders, anxiety disorders, and alcohol dependence using the CIDI (see Table A1 in Appendix). We also used the Bradford Somatic Inventory (Mumford et al., 1991) which provides a cut off indicating likely somatoform symptoms.

**Table A1:** Post-traumatic stress disorder (PTSD) comorbidity (Dorrington et al., 2014).

DSM-IV lifetime diagnosis	*n* (%) in whole population	*n* (%) exposed to trauma	Unadjusted OR (95% CI) exposed to trauma	P	*n* (%) with comorbid PTSD	Unadjusted OR (95% CI) comorbid PTSD	P
Depression	397 (6.6)	217 (10.0)	2.24 (1.82–2.76)	<0.001	44 (37.0)	9.23 (6.27–13.59)	<0.001
Dysthymia	66 (1.2)	52 (2.4)	2.90 (1.85–4.55)	<0.001	17 (14.4)	14.83 (8.41–26.17)	<0.001
Any anxiety disorder	546 (9.1)	270 (12.4)	1.82 (1.52–2.18)	<0.001	46 (40.0)	7.22 (4.89–10.65)	<0.001
Generalised anxiety disorder	239 (4.0)	138 (6.3)	2.49 (1.91–3.26)	<0.001	29 (12.18)	9.16 (5.90–14.22)	<0.001
Agoraphobia	97 (1.6)	50 (2.3)	1.17 (1.06–1.30)	0.002	15 (12.6)	1.79 (1.55–2.07)	<0.001
Social phobia	105 (1.8)	60 (2.8)	2.38 (1.59–3.54)	<0.001	11 (9.24)	6.28 (3.27–12.06)	<0.001
Simple phobia	356 (5.9)	177 (8.1)	1.68 (1.36–2.09)	<0.001	28 (23.5)	5.25 (3.39–8.15)	<0.001
Panic disorder	28 (0.5)	20 (0.9)	4.42 (1.94–10.06)	<0.001	9 (7.6)	25.28 (11.18–57.16)	<0.001
Alcohol dependence⁎⁎	111 (1.9)	66 (3.0)	1.27 (1.16–1.39)	<0.001	14 (11.8)	8.00 (4.38–14.46)	<0.001
Somatisation (BSI* > 12)	388 (6.5)	179 (8.2)	1.55 (1.25–1.92)	<0.001	28 (23.5)	4.74 (3.08–7.31)	<0.001
Any psychiatric disorder	1152 (19.2)	560 (25.8)	1.90 (1.67–2.17)	<0.001	80 (69.6)	10.28 (6.8–15.54)	<0.001

Dorrington, S., Zavos, H., Ball, H., McGuffin, P., Rijsdijk, F., Siribaddana, S., Sumathipala, A., Hotopf, M., 2014. Trauma, post-traumatic stress disorder and psychiatric disorders in a middle-income setting: prevalence and comorbidity. British Journal of Psychiatry 205, 383–389.

⁎ BSI, Bradford Somatic Inventory.a. Moderate depressive disorder.

⁎⁎ Men only as alcohol dependence was absent in women.

### 3.2 Sociodemographic and socioeconomic factors

Sociodemographic (age, sex, ethnicity, twin status, marital status) and socioeconomic factors (years of education, employment and urbanicity) were recorded, and a composite measure of deprivation was created based on environmental measures and household characteristics. Items included: house tenure and type (3 items); overcrowding (1 item); quality of structural materials (3 items); toilet and water facilities (3 items); lighting and fuel type (2 items); household commodities (4 items); access to means of transport (1 item); a subjective report of one's financial situation (1 item); and experiencing hunger due to poverty in the last three months (1 item) (Ball et al., 2010, Siribaddana et al., 2008).

### 3.3 Maladaptive family functioning

The Childhood Experience of Care and Abuse Questionnaire (CECA-Q), is an investigator-based retrospective interview, which was used to measure experiences of maladaptive family functioning before 17 years of age (Smith et al., 2002). Studies of both clinical and community populations have demonstrated the validity of the CECA-Q (Bifulco et al., 2005, Smith et al., 2002). Maladaptive family functioning was measured using 4 scales: parental neglect, parental antipathy, punishment and separation. We used a modified version of the CECA-Q because local experts were concerned about the acceptability of asking about sexual trauma at first contact in a population study.

#### 3.3.1 Parental neglect and antipathy

The CECA-Q parenting scale includes measures of neglect (8 items) and antipathy (8 items) scored for each parent. Each item is scored on a 5 point scale from “definitely” to “not at all”. The neglect score includes items on whether the child was fed and clothed properly as well as parental interest in everyday activities, school work and friendships. The antipathy scale measures the degree of dislike, criticism, hostility or coldness shown to the child by each parent as well as favouritism and scapegoating in comparison with other siblings. The constituent scales (maternal neglect, maternal antipathy, paternal neglect, paternal antipathy) can either be used as continuous scores or as binary variables. We used validated cut off points to generate the latter (Bifulco et al., 2005). In our main analysis we combined maternal and paternal neglect to create one neglect exposure and maternal and paternal antipathy to create one antipathy exposure. In total, 5906 (98.2%) people completed the neglect questionnaire, and 5885 (97.9%) completed the antipathy questionnaire.

### 3.4 Punishment and separation

In addition to measures of neglect and antipathy, the CECA-Q contains a binary question about punishment before age 17: “were you ever hit repeatedly with an implement (such as a belt or stick) or punched, kicked or burnt by someone in the household?” and a binary question about separation “were you ever separated from your mother or father for more than a year before the age of 17?”. Of all participants, 5977 (99.4%) completed the separation question and 5993 (99.7%) completed the punishment question.

### 3.5 Ethics committee approval

The study received approvals from the Institute of Psychiatry, King's College London Research Ethics Committee; the Ethical Review Committee, University of Sri Jayewardanepura; and the World Health Organisation's Research Ethics Committee.

### 3.6 Statistical analysis

Analyses were conducted in Stata version 11, with appropriate account taken of the clustered nature of twin data using the svyset command in stata. We analysed the association between MFFA (parental neglect, separation, punishment and antipathy) and PTSD and other DSM psychiatric diagnosis in the sample exposed to trauma, using logistic regression. Our first model controls for individual demographic variables (sex, age, ethnicity and twin status). The second model includes measures related to socioeconomic status (employment, deprivation, years of education and urbanicity). Our third model controls for covariates from models 1 and 2 plus an additional covariate from our hypothesis, either (a) interpersonal violence (b) cumulative events or (c) other psychopathology.

## 4 Results

### 4.1 Maladaptive family functioning adversities

Table 1 shows the associations between MFFA and socio-demographic variables. 26.5% of people reported exposure to any MFFA. Separation (15.6%) and neglect (7.7%) were more prevalent than experiences of antipathy (4.0%) and punishment (4.2%). The pattern of exposure to MFFA differed according to type of MFFA. Exposure to parental antipathy in childhood was higher amongst women, whereas childhood punishment was highest amongst men. The oldest age-group reported less parental antipathy and less punishment but greater neglect. Those with the highest deprivation scores were exposed to more parental neglect and antipathy. Participants with education over age 10 were less exposed to neglect and separation. Parental neglect was more prevalent in urban settings and both neglect and separation were reported more in ethnic minority groups. Twins were more likely to report separation and punishment than non-twins. Overall 4.2% (247) of participants reported exposure to 2 MFFA and 1.3% (76) were exposed to 3–4 MFFA.

**Table 1:** Distribution of childhood adversities.

Variable	Population	Neglect		Antipathy		Separation		Punishment	
		N(%)	OR (95% CI)	N(%)	OR (95% CI)	N(%)	OR (95% CI)	N(%)	OR (95% CI)
Total	6012	464 (7.7)		240 (4.0)		940 (15.6)		255 (4.2)	
Sex									
Male	2765	205 (7.8)	1	93 (3.5)	1	424 (15.3)	1	152 (5.5)	1
Female	3247	259 (8.6)	1.11 (0.92–1.35)	146 (4.8)	1.38 (1.06–1.80)	516 (15.8)	1.04 (0.89–1.22)	103 (3.2)	0.56 (0.44–0.73)
Age (in quintiles)									
16–23	1285	58 (4.8)	1	54 (4.4)	1	219 (17.0)	1	61 (4.8)	1
24–30	1138	92 (8.5)	1.85 (1.32–2.59)	49 (4.5)	1.02 (0.69–1.52)	213 (11.3)	1.12 (0.87–1.45)	47 (4.1)	0.86 (0.59–1.28)
31–39	1253	94 (7.9)	1.72 (1.23–2.41)	54 (4.6)	1.02 (0.70–1.51)	194 (15.5)	0.90 (0.70–1.15)	70 (5.6)	1.19 (0.83–1.69)
40–51	1200	125 (11.1)	2.48 (1.80–3.42)	53 (4.7)	1.06 (0.72–1.56)	154 (12.8)	0.72 (0.55–0.92)	47 (4.0)	0.82 (0.55–1.21)
52+	1138	95 (9.0)	1.97 (1.41–2.77)	29 (2.8)	0.61 (0.38–0.96)	160 (14.1)	0.80 (0.62–1.03)	30 (2.7)	0.54 (0.35–0.85)
Quintiles deprivation									
Lowest quintile	1198	61 (5.3)	1	34 (3.0)	1	166 (13.9)	1	48 (4.0)	1
2	1205	79 (6.9)	1.32 (0.93–1.86)	49 (4.3)	1.45 (0.93–2.26)	178 (14.8)	1.08 (0.85–1.38)	55 (4.6)	1.15 (0.77–1.70)
3	1202	79 (7.0)	1.35 (0.95–1.90)	43 (3.8)	1.28 (0.81–2.03)	176 (14.6)	1.07 (0.84–1.37)	47 (3.9)	0.98 (0.65–1.47)
4	1202	90 (8.1)	1.57 (1.12–2.20)	48 (4.3)	1.45 (0.92–2.26)	208 (17.3)	1.29 (1.02–1.65)	55 (4.6)	1.15 (0.78–1.71)
Highest quintile	1202	155 (13.6)	2.82 (2.07–3.84)	65 (5.7)	1.97 (1.29–3.01)	212 (17.6)	1.33 (1.04–1.70)	50 (4.2)	1.04 (0.70–1.56)
Employment									
Full time	2646	203 (8.1)	1	103 (4.1)	1	397 (15.0)	1	124 (4.7)	1
Part time/seasonal	381	50 (13.7)	1.81 (1.30–2.52)	23 (6.3)	1.59 (1.00–2.54)	75 (19.7)	1.40 (1.05–1.88)	36 (9.5)	2.13 (1.45–3.14)
Student	567	19 (3.5)	0.42 (0.26–0.68)	25 (4.6)	1.14 (0.73–1.79)	84 (14.8)	0.96 (0.70–1.31)	24 (4.2)	0.90 (0.41–0.77)
Unable to work	145	13 (10.2)	1.29 (0.71–2.33)	7 (5.5)	1.36 (0.62–3.00)	25 (17.2)	1.22 (0.77–1.93)	8 (5.7)	1.22 (0.59–2.55)
Home-maker	2225	175 (8.4)	1.05 (0.85–1.30)	80 (3.9)	0.95 (0.70–1.27)	346 (15.6)	1.05 (0.89–1.23)	60 (2.7)	0.57 (0.41–0.77)
Other	38	4 (10.8)	1.38 (0.49–3.95)	1 (2.8)	0.67 (0.09–4.95)	14 (36.8)	3.44 (1.67–7.07)	3 (7.9)	1.74 (0.53–5.74)
Marital status									
Married	3572	317 (9.4)	1	141 (4.2)	1	557 (15.5)	1	145 (4.1)	1
Single	2061	114 (5.9)	0.61 (0.49–0.76)	78 (4.0)	0.96 (0.73–1.28)	330 (16.0)	1.03 (0.88–1.22)	95 (4.6)	1.15 (0.88–1.49)
Previously married	375	33 (9.6)	1.03 (0.71–1.50)	20 (5.8)	1.42 (0.88–2.31)	53 (14.1)	0.89 (0.66–1.21)	15 (4.0)	0.99 (0.57–1.70)
Years of education									
Up to 10	2114	220 (11.2)	1	94 (4.8)	1	369 (17.5)	1	110 (5.2)	1
11–12	1691	117 (7.3)	0.62 (0.49–0.79)	58 (3.6)	0.75 (0.54–1.05)	260 (15.4)	0.86 (0.71–1.03)	54 (3.2)	0.60 (0.43–0.83)
12+	1802	110 (6.4)	0.54 (0.42–0.68)	71 (4.1)	0.86 (0.63–1.18)	257 (14.3)	0.79 (0.65–0.95)	80 (4.4)	0.84 (0.63–1.13)
Urbanicity									
Semi urban	3657	245 (7.1)	1	151 (4.4)	1	541 (14.8)	1	159 (4.4)	1
Urban	2355	219 (9.8)	1.42 (1.17–1.72)	88 (4.0)	0.90 (0.69–1.17)	399 (16.9)	1.18 (1.00–1.39)	96 (4.1)	0.93 (0.72–1.21)
Ethnicity									
Sinhala	5556	404 (7.7)	1	214 (4.1)	1	843 (15.2)	1	236 (4.3)	1
Non-sinhala	458	60 (13.9)	1.92 (1.44–2.57)	25 (5.8)	1.44 (0.94–2.20)	97 (21.2)	1.49 (1.11–2.01)	19 (4.2)	0.97 (0.60–1.57)
Twin status									
Singleton	2019	173 (8.9)	1	72 (3.7)	1	286 (14.2)	1	66 (3.3)	1
Twin	3995	291 (7.8)	0.86 (0.71–1.05)	167 (4.5)	1.21 (0.91–1.61)	654 (16.5)	1.20 (1.02–1.41)	189 (4.8)	1.47 (1.11–1.96)
Interpersonal violence									
No exposure	4813	366 (7.6)	1	191 (4.0)	1	748 (14.8)	1	178 (3.5)	1
Exposure	856	98 (11.5)	1.57 (1.24–1.99)	48 (5.6)	1.44 (1.04–2.00)	191 (21.1)	1.55 (1.29–1.86)	77 (8.5)	2.55 (1.91–3.40)
Cumulative trauma									
0	3828	258 (7.2)	1	131 (3.6)	1	524 (13.8)	1	88 (2.31)	1
1	1393	108 (8.2)	1.15 (0.91–1.46)	51 (3.9)	1.07 (0.76–1.49)	233 (16.8)	1.26 (1.06–1.50)	79 (5.7)	2.54 (1.82–3.55)
2+	787	97 (13.1)	1.96 (1.52–2.52)	56 (7.6)	2.17 (1.56–3.03)	179 (22.9)	1.86 (1.53–2.26)	88 (11.2)	5.32 (3.84–7.38)

### 4.2 Maladaptive family functioning and trauma exposure

Table 1 shows that each domain of maladaptive family functioning was associated with greater reported trauma exposure – both to interpersonal violence, and to cumulative exposure to trauma. Table 2 explores the associations between maladaptive family functioning and any trauma exposure. Parental neglect, antipathy, punishment, separation and multiple MFFA exposure were all associated with trauma exposure, punishment having the strongest association. Controlling for sociodemographic and socioeconomic factors did not reduce the association between individual MFFA and trauma exposure, suggesting that the association between individual maladaptive family functioning and trauma is not explained by confounding due to demographic or economic status. The association between multiple MFFA and trauma was attenuated in model 2, by socioeconomic variables.

**Table 2:** The association between MFFA and trauma exposure.

	Population N (%)	Trauma exposure odds ratio (95% CI) (unadjusted)	Trauma exposure odds ratio (95% CI) model 1	Trauma exposure odds ratio (95% CI) model 2
Neglect	464 (8.2)	1.43 (1.18–1.74) *p* < 0.001	1.46 (1.19–1.77) *p* < 0.001	1.45 (1.18–1.78) *p* < 0.001
Antipathy	239 (4.2)	1.45 (1.11–1.90) *p* = 0.006	1.55 (1.17–2.04) *p* = 0.002	1.61 (1.20–2.16) *p* = 0.001
Punishment	255 (4.3)	3.51 (2.63–4.67) *p* < 0.001	3.33 (2.48–4.47) *p* < 0.001	3.38 (2.49–4.58) *p* < 0.001
Separation	940 (15.6)	1.47 (1.26–1.70) *p* < 0.001	1.50 (1.29–1.74) *p* < 0.001	1.53 (1.31–1.78) *p* < 0.001
Any MFFA	1544 (25.7)	1.49 (1.31–1.68) *p* < 0.001	1.49 (1.32–1.69) *p* < 0.001	1.54 (1.35–1.75) *p* < 0.001
Number of MFFA				
1	1221 (20.3)	1.36 (1.19–1.55) *p* < 0.001	1.36 (1.19–1.56) *p* < 0.001	1.40 (1.22–1.61) *p* < 0.001
2	247 (4.1)	1.91 (1.46–2.50) *p* < 0.001	1.95 (1.48–2.57) *p* < 0.001	2.06 (1.56–2.73) *p* < 0.001
3+	76 (1.3)	2.65 (1.65–4.25) *p* < 0.001	2.64 (1.61–4.34) *p* < 0.001	2.59 (1.57–4.30) *p* < 0.001

Model 1 age, sex, ethnicity and twin status.

Model 2 model 1 variables plus deprivation, employment, marital status, years of education and urbanicity.

### 4.3 Maladaptive family functioning and PTSD in the trauma exposed population

Table 3 shows the associations between maladaptive family functioning and PTSD in the trauma-exposed population. All measures of maladaptive family functioning except separation and multiple MFFA were associated with PTSD after controlling for sociodemographic factors (models 1 and 2). Controlling for socio-demographic and economic status had little impact on effect sizes. Controlling for interpersonal violence (model 3a), cumulative trauma (3b) or psychiatric disorder (3c) attenuated effect sizes, however the effects remained significant for antipathy and punishment with substantial odds ratios > 2).

**Table 3:** PTSD and other psychiatric diagnosis in trauma exposed population by MFFA.

PTSD diagnosis		Population percent exposed to trauma	N(%) with PTSD	PTSD odds ratio (95% CI) (unadjusted)	PTSD odds ratio (95% CI) model 1	PTSD odds ratio (95% CI) model 2	PTSD odds ratio (95% CI) Model 3A	PTSD odds ratio (95% CI) Model 3B	PTSD odds ratio (95% CI) Model 3C
	Neglect	44.3	21 (10.3)	2.21 (1.34–3.64) p = 0.002	2.17 (1.32–3.59) p = 0.002	2.09 (1.24–3.52) p = 0.005	1.80 (1.05–3.08) p = 0.03	1.75 (1.02–3.01) p = 0.04	1.60 (0.92–2.77) p = 0.1
	Antipathy	45.0	15 (14.3)	3.15 (1.75–5.65) *p* < 0.001	2.86 (1.59–5.14) *p* < 0.001	2.80 (1.51–5.21) *p* = 0.001	2.25 (1.20–4.21) *p* = 0.01	2.18 (1.14–4.17) *p* = 0.02	2.01 (1.04–3.90) *p* = 0.04
	Punishment	65.5	19 (11.5)	2.47 (1.47- 4.14) *p* < 0.001	2.67 (1.57–4.52) *p* < 0.001	2.63 (1.53–4.52) *p* < 0.001	2.01 (1.14–3.56) *p* = 0.02	2.06 (1.16–3.65) *p* = 0.01	2.28 (1.29–4.03) *p* = 0.005
	Separation	44.0	27 (6.6)	1.27 (0.82–1.98) *p* = 0.3	1.28 (0.82–2.01) *p* = 0.3	1.28 (0.81–2.02) *p* = 0.3	1.19 (0.75–1.89) *p* = 0.5	1.09 (0.69–1.74) *p* = 0.7	1.13 (0.70–1.83) *p* = 0.6
	Any MFFA	43.5	57(8.5)	2.66 (1.83–3.85) *p* < 0.001	2.49 (1.56–3.98) *p* < 0.001	2.67 (1.64–4.38) *p* < 0.001	2.46 (1.48–4.08) *p* = 0.001	2.01 (1.18–3.43) *p* = 0.01	2.22 (1.34–3.67) *p* = 0.002
	No of MFFA								
	1	41.3	36 (7.2)	2.10 (1.38–3.20) *p* = 0.001	2.14 (1.28–3.56) *p* = 0.004	2.30 (1.35–3.92) *p* = 0.002	2.13 (1.23–3.69) *p* = 0.007	1.90 (1.06–3.40) *p* = 0.03	1.95 (1.12–3.37) *p* = 0.02
	2	49.8	13 (10.6)	3.84 (2.07–7.12) *p* < 0.001	3.18 (1.45–7.01) *p* = 0.004	3.42 (1.50–7.80) *p* = 0.003	2.99 (1.28–6.97) *p* = 0.01	2.07 (0.85–5.02) *p* = 0.1	2.97 (1.29–6.86) *p* = 0.01
	3+	57.8	8 (18.6)	8.26 (3.79–17.99) *p* < 0.001	7.16 (2.37–21.66) *p* < 0.001	6.94 (2.32–20.75) *p* = 0.001	6.64 (2.17–20.31) *p* = 0.001	3.16 (0.74–13.63) *p* = 0.12	3.36 (1.13–11.71) *p* = 0.03
Other DSM psychiatric diagnosis		Population percent exposed to trauma	N(%) with other DSM	Other DSM odds ratio (95% CI) (unadjusted)	Other DSM odds ratio (95% CI) model 1	Other DSM odds ratio (95% CI) model 2	Other DSM odds ratio (95% CI) Model 3A	Other DSM odds ratio (95% CI) Model 3B	Other DSM odds ratio (95% CI) Model 3C
	Neglect	44.3	82 (40.4)	2.15 (1.59–2.91) *p* < 0.001	2.07 (1.52–2.81) *p* < 0.001	1.81 (1.30–2.50) *p* < 0.001	1.67 (1.20–2.32) *p* = 0.002	1.65 (1.19–2.30) *p* = 0.003	1.67 (1.19–2.33) *p* = 0.003
	Antipathy	45.0	51 (48.1)	2.88 (1.91–4.33) *p* < 0.001	2.66 (1.76- 4.01) *p* < 0.001	2.74 (1.79–4.20) *p* < 0.001	2.44 (1.60–3.72) *p* < 0.001	2.35 (1.53- 3.60) *p* < 0.001	2.39 (1.53–3.74) *p* < 0.001
	Punishment	65.5	60 (36.1)	1.70 (1.22–2.39) *p* = 0.002	1.87 (1.34–2.63) *p* < 0.001	1.80 (1.26–2.55) *p* = 0.001	1.59 (1.12–2.27) *p* = 0.01	1.60 (1.12–2.30) *p* = 0.01	1.61 (1.11–2.35) *p* = 0.01
	Separation	44.0	118 (28.8)	1.20 (0.94- 1.53) *p* = 0.1	1.23 (0.96–1.57) *p* = 0.1	1.17 (0.90–1.51) *p* = 0.2	1.14 (0.88–1.47) *p* = 0.3	1.09 (0.83–1.42) *p* = 0.5	1.16 (0.89–1.50) *p* = 0.3
	Any MFFA	43.5	194 (37.3)	1.71 (1.48–1.98) *p* < 0.001	1.67 (1.39–2.01) *p* < 0.001	1.56 (1.28–1.89) *p* < 0.001	1.52 (1.25–1.85) *p* < 0.001	1.43 (1.18–1.74) *p* < 0.001	1.46 (1.20–1.78) *p* < 0.001
	No of MFFA								
	1	41.3	130 (25.9)	1.52 (1.30–1.79) *p* < 0.001	1.52 (1.24–1.86) *p* < 0.001	1.44 (1.17–1.78) *p* = 0.001	1.41 (1.14–1.75) *p* < 0.001	1.36 (1.09–1.69) *p* = 0.006	1.37 (1.11–1.70) *p* = 0.004
	2	49.8	40 (32.8)	1.88 (1.38–2.56) *p* < 0.001	1.80 (1.24–2.62) *p* = 0.002	1.52 (1.02–2.28) *p* = 0.04	1.47 (0.98–2.20) *p* = 0.06	1.33 (0.89–1.98) *p* = 0.2	1.39 (0.92–2.12) *p* = 0.1
	3+	57.8	24 (54.5)	5.64 (3.56–8.92) *p* < 0.001	5.33 (2.93–9.70) *p* < 0.001	5.00 (2.64–9.46) *p* < 0.001	4.81 (2.52–9.17) *p* < 0.001	4.00 (2.06–7.76) *p* < 0.001	4.28 (2.14–8.53) *p* < 0.001

Model 1 age, sex, ethnicity and twin status;

Model 2 model 1 variables plus deprivation, employment, marital status, years of education and urbanicity;

Model 3A includes model 1 and 2 variables plus high risk events (interpersonal violence); Model 3B includes model 1 and 2 variables plus cumulative trauma;Model 3C includes model 1 and 2 variables plus psychiatric diagnoses (for analyses where PTSD is dependent variable) or PTSD (for analysis where psychiatric diagnosis was dependent variable).

### 4.4 Maladaptive family functioning and other DSM psychiatric diagnosis in the trauma exposed population

Table 3 also shows that for the trauma-exposed group, punishment, parental neglect and antipathy were strongly associated with other psychiatric diagnoses in all models. Neglect, antipathy, punishment and multiple MFFA continued to be associated with other DSM psychiatric diagnosis in the fully adjusted model. Antipathy had the strongest fully adjusted association with other DSM psychiatric diagnosis. Separation had no association with any psychiatric diagnosis in unadjusted or adjusted models. The association between other psychiatric diagnosis and MFFA persisted for neglect, antipathy, punishment and multiple MFFA after controlling for cumulative trauma, high risk trauma, and PTSD.

## 5 Discussion

In summary, MFFA was strongly associated with trauma exposure, and moderated the association between trauma exposure and both PTSD and other DSM psychiatric diagnosis. This was not explained by interpersonal violence, cumulative trauma exposure or other psychopathology.

We have demonstrated that childhood adversity is not randomly distributed. Consistent with previous research, we found neglect, antipathy and separation to be associated with higher levels of deprivation (Pearlin et al., 2005, Thoburn et al., 2000). Similar variations in exposure to childhood adversity have been documented within countries and across high, middle and low income countries, but population level data from within lower and middle income countries is sparse and based on different measures of adversity (Norman et al., 2012, Rosenman and Rodgers, 2004).

We found strong evidence in support of our first hypothesis that MFFA is associated with greater exposure to traumatic events and that this was not explained by socioeconomic factors measured in adult life. It seems, therefore, that individuals with more troubled upbringings have greater exposure to all trauma, trauma involved in inter-personal violence and multiple trauma, than those without such childhood experiences. The fact that the odds ratios between individual MFFA and trauma exposure were barely affected by controlling for multiple socioeconomic and demographic variables implies that the association is not accounted for by incomplete adjustment of confounding. Instead, it suggests that there is a more direct relationship between MFFA and later traumas. This study offers much needed evidence about notable types of stress proliferation results in increased vulnerability to PTSD (Pearlin et al., 2005).

This is further supported in the evidence we find supporting our second hypothesis, that MFFA increases vulnerability to PTSD when traumatic events occur*.* For the population exposed to traumatic events, those reporting MFFA had more PTSD. When type and dose of trauma and psychiatric comorbidity were controlled for, the association between antipathy, punishment and PTSD persisted. The associations were particularly striking for those experiencing multiple MFFA. These findings are consistent with the results of a meta-analysis based on studies predominantly from high income countries which observed significant associations with post traumatic stress disorder (PTSD) and childhood physical abuse (Norman et al., 2012).

Researchers have explored the physiological and psychological impact of childhood adversity. Results suggest that early adversity can affect conceptual processing of trauma and alter the meaning of adult trauma (Ehlers and Clark, 2000), leading to negative appraisals and a greater sense of threat at the time of trauma exposure, both risk factors for PTSD (Brewin et al., 2000, Iversen et al., 2007, Ozer et al., 2003). Child maltreatment also has a long term impact on functional and structural imaging markers associated with PTSD; examples include limbic hyper responsiveness to aversive stimuli, and reduced hippocampus grey matter volume (Dannlowski et al., 2012). Biological mechanisms found to be involved in PTSD include disruption of immune responses (Altemus et al., 2003, Danese et al., 2007, Fagundes et al., 2013), or the hypothalamic-pituatry- adrenal axis and catecholamines (Young and Breslau, 2004). These abnormalities are also seen in people who report early adversity (Danese et al., 2007, Heim et al., 2000).

MFFA is associated with other DSM psychiatric disorders, confirming the established association between MFFA and psychopathology (Collishaw et al., 2007, Hughes et al., 2017, Kessler et al., 2010, Ni et al., 2015). In a previous study of this community sample we found panic disorder to be the psychiatric disorder most strongly associated with trauma exposure (Dorrington et al., 2014). A meta-analysis of psychopathology and early maltreatment, observed associations between panic and childhood physical abuse (Norman et al., 2012). However, our results do not strongly support our third hypothesis, that MFFA acts on PTSD risk via prior pre-trauma psychiatric disorders, as controlling for psychiatric comorbidities had little impact on the MFFA-PTSD association. We found similar effect sizes for the impact of childhood adversity on other psychiatric disorders, which persisted after controlling for socio-economic variables, cumulative trauma and PTSD.

## 6 Strengths and limitations

In this large study we carefully ascertained a population sample and had exceptionally high participation rates. However, the cross-sectional nature of the data limit conclusions on the direction of causation. The twin status of our participants is unlikely to have had a material impact on these results and twin status was controlled for in the analysis. An additional limitation is that traumatic events might have occurred at the same age or even before MFFA. Because of concerns that participants would be uncomfortable answering questions about childhood sexual abuse, this was not included in our ascertainment of early adversity, and therefore our measurement of MFFA is incomplete. There is strong evidence that childhood sexual abuse is associated with mental disorders (Castellvi et al., 2017, Devries et al., 2014, Fry et al., 2012, Mandelli et al., 2015), it is likely that its inclusion would have a similar or stronger mediating effect than other measures of MFFA. There is nothing to suggest, however, that our main findings would have been changed by its inclusion.

## 7 Conclusions

The excess PTSD in people with childhood adversity may partially be explained by greater subsequent trauma exposure, but not by trauma severity or comorbid psychiatric disorder. This suggests a specific relationship between childhood adversity and subsequent vulnerability to PTSD. Future studies may want to take into account the increased exposure to trauma across the life course amongst patients with MFFA.

## Declaration of interest

The research was funded by Wellcome Trust. This paper represents independent research part-funded by the National Institute for Health Research (NIHR) Biomedical Research Centre at South London and Maudsley NHS Foundation Trust and King's College London. The views expressed are those of the authors and not necessarily those of the NHS, the NIHR or the Department of Health.

## Author's contributions

SD and MH wrote the paper. SS assisted in the design of the study and was the main study coordinator. He trained and managed the field workers, and was responsible for data collection and quality control; HB cleaned the data; SD and HZ analysed the data; AS is the Principal Investigator in Sri Lanka, and contributed to the establishment of the twin registry, study design, and management of the study; PMcG contributed to the design of the study; MH is Principal Investigator in the UK, with responsibility for the design of the study. With AS he secured funding for the study, coordinated the wider study group, and supervised the local team in practical issues related to study design and data collection. All authors commented on drafts of the paper.

## Conflict of interest statement

Authors have no conflict of interest to declare.

## Appendix B Supplementary materials

Image, application 1
